# INDUCIBLE SKELETAL MUSCLE SOD2 KNOCKDOWN MIMICS AGE-RELATED MITOCHONDRIAL DYSFUNCTION IN MOUSE SKELETAL MUSCLE

**DOI:** 10.1093/geroni/igae098.2626

**Published:** 2024-12-31

**Authors:** Ethan Ostrom, Sahir Sandhu, David Marcinek

**Affiliations:** University of Washington, Seattle, Washington, United States; University of Washington, Seattle, Washington, United States; University of Washington, Seattle, Washington, United States

## Abstract

Aging causes declines in mitochondrial energetic efficiency and increased reactive oxygen species (ROS) production in skeletal muscle, leading to declines in muscle function and impaired stress resilience. While perturbations in redox homeostasis have been associated with declines in muscle and mitochondrial function, the mechanisms and time course of this decay have not been established. Here we use a new model of inducible skeletal muscle specific knockdown of SOD2 (KD) to produce an oxidative stress and compare the functional declines to those seen in aging muscle mitochondria. We demonstrate the model induces skeletal muscle specific SOD2 protein KD by 50% at 3 weeks and 90% decrease by 8 weeks. KD showed a significant decline in in vivo muscle force in female KD mice (p=0.0249), but not male KD mice compared to controls at eight weeks. KD resulted in significant reductions in aconitase 2 (ACON2) activity an enzyme sensitive to oxidative stress. Similar to aged skeletal muscle, KD showed significant deficits of complex I-driven respiration (p< 0.0001), max oxphos capacity (p=0.0001), and maximal ROS production rate (p< 0.05) in permeabilized muscle fibers. Interestingly, the CI impairment in SOD2 KD was dependent the order of substrate addition indicating that SOD2 KD affects metabolic flexibility of the mitochondria. These results show that short term redox stress reproduces some aspects of aged skeletal muscle mitochondria. Ongoing experiments are testing the metabolic and molecular mechanisms underlying impaired metabolic flexibility related to elevated redox stress in the SOD2 KD and their relevance to aging muscle.

